# A how-to guide for code sharing in biology

**DOI:** 10.1371/journal.pbio.3002815

**Published:** 2024-09-10

**Authors:** Richard J. Abdill, Emma Talarico, Laura Grieneisen

**Affiliations:** 1 Section of Genetic Medicine, Department of Medicine, University of Chicago, Chicago, Illinois, United States of America; 2 Department of Biology, University of British Columbia—Okanagan Campus, Kelowna, British Columbia, Canada; 3 Okanagan Institute for Biodiversity, Resilience, and Ecosystem Services, University of British Columbia—Okanagan Campus, Kelowna, British Columbia, Canada

## Abstract

In 2024, all biology is computational biology. Computer-aided analysis continues to spread into new fields, becoming more accessible to researchers trained in the wet lab who are eager to take advantage of growing datasets, falling costs, and novel assays that present new opportunities for discovery. It is currently much easier to find guidance for implementing these techniques than for reporting their use, leaving biologists to guess which details and files are relevant. In this essay, we review existing literature on the topic, summarize common tips, and link to additional resources for training. Following this overview, we then provide a set of recommendations for sharing code, with an eye toward guiding those who are comparatively new to applying open science principles to their computational work. Taken together, we provide a guide for biologists who seek to follow code sharing best practices but are unsure where to start.

## Abstract 2

For those who want to share their code but don’t know where to start, this Essay distils dozens of articles on reproducibility and research software, collecting the most important practical details of how to provide computational transparency even if you aren’t a trained software developer.

## Introduction

Reproducible computational practices, and open science more broadly, are the subject of many discussions about competing priorities: Transparency is good, but its implementation is time intensive and poorly incentivized [1]. Open research is associated with more citations and more media coverage [2], but it can expose researchers to new avenues for harassment and suppression [3]. There are also many resources promoting particular approaches to performing computational work [4,5] and developing research software [6]. But amidst the discussions of how to perform computational research, where to publish it, and how to organize your files, there is a dearth of information on how to be transparent about work that’s already been done, particularly for biologists who may not specialize in computational work. Many researchers are unsure how to share code or, as a recent Springer Nature survey found, even where to upload their data [7].

A complicating factor is the wide variation between fields in the standards for data and code sharing, as well as the types of data sets and code used. For example, data sharing standards are well reviewed in the ecological literature [8,9], and major journals in the field of ecology have extensive data sharing policies, such as Ecological Society of America’s Open Research Policy “Definitions” page [10]. However, these practices are less standardized in other fields of biology. This means that research and journals at the intersection of multiple fields—such as microbiome science, which integrates medicine, ecology, and computational biology—may not have scientists trained in uniform standards of data and code sharing. Finally, many established papers on best practices focus on how to design a study from the outset to fit into a reproducible science framework. However, science is messy, and as projects and data sets are passed between lab members, the researchers who are assembling the final paper for submission often inherit files and code that were not created using best practices.

The goal of our paper, therefore, is to provide an integrative guide for sharing code, such that these practices can be implemented across biological subfields and stages of the research process. We focus on the implementation details particular to reproducible results—those that can be regenerated using the original data—as opposed to replicable results, in which another group is able to draw similar conclusions from new data [11,12].

We can all share code, if not because we want someone else to be able to use it in the future, then at least because we have already used it for the research being reported. The code used to analyze data, perform statistical tests, and build visualizations is no less critical to a project than reagents used at the bench and should be disclosed for the same reasons: In addition to providing critical information about the conditions under which the study was performed, sharing this information enables others to more easily validate computationally derived conclusions. It also reduces the effort required to apply similar methods in new projects by providing an example that allows others to avoid issues you may have already solved. A 2023 study across dozens of participants from 13 countries found broad support for publications that clearly state whether code was shared openly, with a persistent identifier (such as a digital object identifier (DOI)) and a clear license [13]. Sharing code also helps protect us against what Donoho and colleagues [14] called “the ubiquity of error” at the heart of the scientific method, which drives scientists to expend effort primarily “in recognizing and rooting out error”: Even the most diligent scientists can miss a typo in a command or misunderstand parameters of a complex function in a package developed elsewhere. Sharing this code—essentially a fine-grained addendum to a methods section—can help bring these issues to the surface and help future researchers avoid them.

In this discussion, we use the term “code” as shorthand for any of the documents interpreted by a computer to generate information used in your manuscript. That covers software you’ve developed to perform analyses (a new algorithm implementation, for example), but it also includes commands used to perform statistical tests and the scripts used to generate figure panels. Most manuscripts don’t come bundled with an entirely new software application, but many—especially those that include the analysis of genomic sequencing data—required code to get their results.

Below, we outline how to build a more easily reproducible project, share a set of resources for improving your coding skills, describe which pieces of your project are most important for others, and lay out how to get your work online in a practical format. Our recommendations were developed as guidance mainly to those who are preparing to share completed work. However, much of this will be easier—and more effective—if a project is started with reproducibility in mind. If you’ve already skipped some of these steps, or inherited a project that was set up in a different way, there is still plenty you can share to provide transparency and demonstrate your process to others.

### Setting up a reproducible project

First things first: Your code is good enough to share [15]! It may be messy and disorganized and cobbled together by self-taught coders who are writing just enough code to get the job done, but you aren’t the only person for whom that’s true. If you trust the code enough that you’ve written a paper about its results, it’s certainly worthy of sharing. Scientific code can be split into 2 broad categories: products that are intended to be reused by others (such as a new software package), and products that aren’t. The preparation, packaging, and archiving of code differs greatly between these 2 categories, and this paper deals mostly with the latter—code shared to demonstrate the computational approach to a single paper, but that others should not expect to work like a broadly applicable tool with a friendly user interface. The recommendations here describe considerations that are helpful, but if some are not practical to implement, it’s important to note that whatever you can share is almost always better than sharing nothing.

There are many guides for reproducible computational biology available online, both peer reviewed and independently published (for example, [9]), covering diverse topics such as telling stories with computational notebooks [16,17], organizational recommendations for large computational projects [18], and standards and checklists that provide very specific examples [19,20]. Rather than duplicate this effort, here we review common recommendations, going from general to specific. We highlight these as examples in the broad categories of accessibility, organization, and minimizing repeated work, with a focus on changes that are particularly impactful but that do not require significant new technical skills. Because this work is focused mostly on sharing projects that are already complete, we urge readers to investigate implementation details for their new projects in the papers and software documentation referenced below.

The first key to sharing your code is to **use code in the first place**. While many tools simplify their operation by enabling users to interact with them using graphics—such as icons, text boxes, and menus—this point-and-click approach can be difficult to document and even more difficult to replicate. Though it can be tempting to skip automation and programmatic approaches in favor of ad hoc point-and-click solutions, these shortcuts can backfire later when trying to repeat an analysis, recall who did what, which subset of the data was used for a figure panel, or exactly which version of a program was used for an important computational step. Understanding command-line tools and their parameterization can be a challenge, but running these operations a second time is much more straightforward than carefully following step-by-step instructions on which buttons to click in which order [21].

#### Keep humans in mind

**Fig 1:**
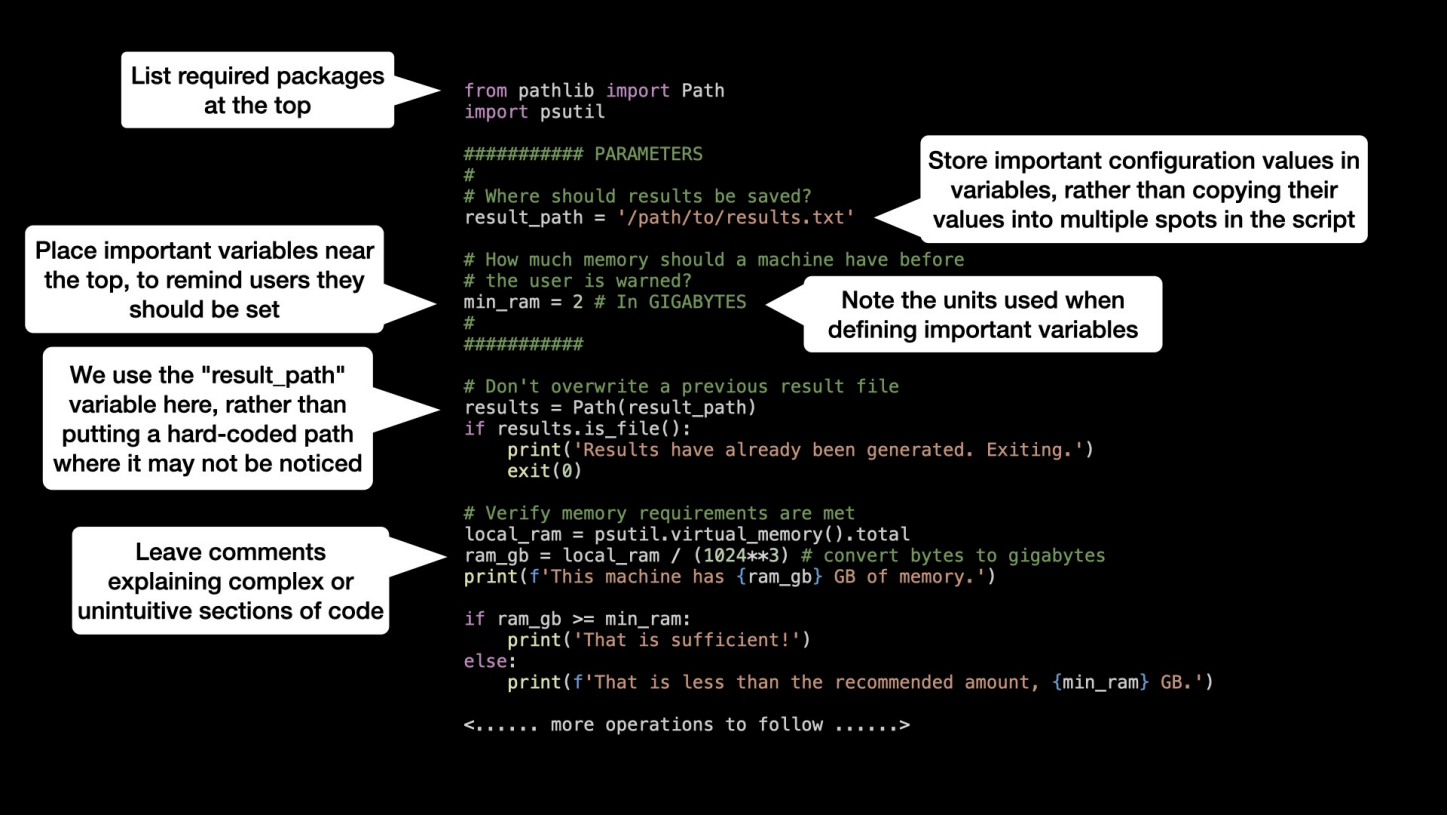
Example code for a reproducible project. The first lines of a longer analysis script written in Python 3 with examples of practices that make the code easier for other users to understand.

Code style and organization is a recurring theme in the literature. In short, consider approaching your code as if it were intended to be read, rather than executed [22]. Give variables helpful names, rather than “foo” or “x,” and leave plenty of comments to explain what different sections of code are doing and reasons for unconventional design decisions that users may be tempted to modify (for examples, see Fig 1) [21].

#### Use a consistent directory structure

Make it easy to distinguish raw data from intermediate files and final results [19,23,24]. For example, when manipulating your data specifically for a visualization, save the version of the data that’s actually displayed [25]: This will be a more convenient file for readers to evaluate and makes it possible to rebuild your figure by simply reloading that file, rather than having to load the original dataset and performing all the processing steps again. Minimize the manual intervention required to run your scripts, and ideally organize them in a way that allows users to run them from start to finish [20]. Provide a “README” file that, at a minimum, walks a user through the intended execution of your code and documents the key steps [26].

#### Don’t be afraid of the tedious work of automating your data cleaning

In particular, avoid manual editing of intermediate files [9,24,25,27]. For example, if script A processes raw data into a table of gene expression levels, and script B summarizes this table by pathway, we should be concerned if there is a step after script A that requires a user to open the table and edit fields by hand to prepare the data for script B. Such quick fixes can be tempting, but they also add risk: A researcher could easily add a typo, corrupt a file in unexpected ways [28], or forget the step altogether.

#### Minimize cut-and-paste errors

Use custom functions to do repeated operations [9], and store important values in prominently commented variables, rather than hard-coding (Glossary) them somewhere deep in the script where a future user may not notice it (Fig 1). For example, if you write out a multistep process that prepares data for a particular visualization but find out later that you need to perform the same steps for a different subset of data in a new figure, avoid copying that code and pasting it farther down in your analysis script. If you later find an error in this code or simply modify it to change a threshold or reorganize the output, it’s easy to forget to scroll back down and change it in two (or more!) places. Repetitive code is one example of “code smell”: code that may work as intended but is suggestive of a larger design flaw that may cause hard-to-find bugs or make it more complicated to modify the program in the future [29]. Organizations and open-source (Glossary) communities have published “style guides” in many popular languages including R (https://style.tidyverse.org) and Python (https://peps.python.org/pep-0008/) that may help avoid some of these issues, but eventually you will develop a “nose” for intuiting when you may be wandering down an ill-advised path.

**Glossary**

**Application Programming Interface (API) keys**

Authentication parameters generally used in a similar manner as passwords when 2 software applications communicate with each other, typically over the internet.

**Command-line utilities**

Computer applications that are guided using text entered into a terminal. Implicitly refers to programs running on the Linux operating system.

**Dependencies**

In the context of software packages or scripts, dependencies are libraries and packages of third-party code that must be present for a given package or script to function.

**Hard-coding**

When the value of a variable or process is manually specified in a way that is not easily modified by someone who wants to run the code. A “hard-coded” file path would only look in one specific directory for a file, rather than exposing a way for the path to be modified via configuration files or command-line options.

**Linux**

Generally, this refers to the family of operating systems that used the Linux kernel. Ubuntu is one such operating system (or “distribution”), as is Rocky Linux, Debian, and Android. High-performance computing clusters rely heavily on nodes running Linux operating systems in the same way many desktop computers run Windows.

**Open-source**

“Source” is a reference to a given software’s “source code,” or the text documents written in languages such as Python or Java that are then prepared and interpreted by the computer for execution. In a general sense, code that is “open” is freely available for inspection, but open-source advocates frequently incorporate additional licensing considerations when deciding whether software is “open,” such as free redistribution of the software [30].

**Software container**

A self-contained computing environment that can be launched with a predefined set of files and software. Similar to virtual machines, containers can be useful in situations where an analysis requires software that is complicated to install or requires very specific system specifications [31].

These are relatively uncontroversial recommendations to make your project easier to manage and adhere more closely to programming best practices, but there are many other opportunities to improve. A 2014 survey showed that software developers reviewing code from computational biology papers were shocked at its content, confusing structure, and lack of documentation [32]. Still, optimizing processes past a point of practical reproducibility may not be worthwhile [33], particularly when competing priorities leave researchers with few professional incentives to tackle the time-consuming work of sharing digital materials.

There are many other recommendations that appear in the literature even without expanding your search beyond papers focused on computational biology: Version control and code review are frequent topics of discussion [9,21,24,34–39], as is “defensive programming” [22], that is, performing what may feel like excessive validation of the inputs and outputs of functions to make sure unexpected states (such as a negative quantity of items) are detected before they can cause problems. For particularly complicated operations, automated testing of sections of code with known inputs and expected outputs is another practice that can make your code more robust, to catch scenarios where a small change has unintended effects elsewhere [40]. These are all complex processes with an intimidating learning curve, but there have never been more resources available for those looking to implement them. Below is a list of valuable websites offering lessons in software development practices applicable to computational biology (Table 1).

**Table 1:** Programming resources.

Program	Description	URL
The Carpentries workshops	Software Carpentry workshops are held all over the world and online. Data Carpentry workshops are less frequent but may be more applicable.	https://carpentries.org
The Carpentries online resources	Carpentries volunteers, many of them full-time researchers, have also built interactive lessons that can be taken at your own pace.	https://carpentries-lab.github.io/good-enough-practices/index.html https://datacarpentry.org/semester-biology/
Glittr	This website organizes hundreds of free, open-source training courses in bioinformatics, from foundational “Python for data analysis” materials to more specific courses in machine learning packages. See the “Reproducibility” topic category in particular.	https://glittr.org
Stack Overflow	A question-and-answer website about computer code. If you have a specific question about anything code related, someone has probably already asked and answered it on Stack Overflow.	https://stackoverflow.com
Biostar	A bioinformatics-focused forum with more than a decade of archived discussions [41].	https://biostars.com
Nextflow and Snakemake	Examples of popular workflow management tools with curated collections of pipelines that may provide the functionality you need, or at least provide a sophisticated example of officially endorsed implementations that you can modify.	Nextflow: https://nf-co.re [42] Snakemake: https://snakemake.github.io/snakemake-workflow-catalog/
GitHub Skills	The “Introduction to GitHub” course is a useful introduction to version control on the most popular platform for open-source code. Other courses cover more advanced concepts, such as pull requests and automation.	https://skills.github.com

### Files to share

When your project is complete and you’re preparing to share your computational work, even the best-organized projects can be a tangled web of intermediate files and quick fixes. In short, you should try to share anything you created that would be necessary to reproduce your calculations. More specifically:

1. **Scripts for data-cleaning and analysis.** Nothing is too mundane! These steps make it easier for others to reproduce your work and can clarify exactly what was done and, crucially, in what order. For example, it may seem trivial to share the exact Python statement you used to perform a straightforward logistic regression, but the “LogisticRegression” function from the popular Python package scikit-learn defaults to a technique that penalizes coefficients in large models (known as L2 regularization) [43,44], while R’s built-in “glm” function doesn’t include similar penalties even as an option [45]. These penalties can dramatically alter the results of a regression, which is why it’s critical for users to understand which options are being used by their statistical libraries. This is just one of many potentially important details about model development [46] that may not be obvious from looking at the outcome but can be tracked down using the original code.
2. **Data visualization code.** Sharing the code you used to generate your figure panels can help people who want to visualize their data in a similar way and clarify finer points of the figure that have been omitted from the legend, intentionally or not. The code can also show exactly how data were filtered and modified before visualization. This may enable readers to explore your results using “living figures” they can modify to look at different subsets of your data, or, in some cases, even add data of their own [47]. It may also serve as valuable documentation for your own future reference.
3. **Parameters used to configure and launch command-line utilities.** Many computational biology tools are executed from the Linux command line (Glossary), with relevant parameters included directly in the command. These parameters may specify the location of input and output files, for example, or set other configuration values such as thresholds or file formats. Ideally, a single script could be executed to run each command and perform your entire analysis process [20]. But even if that isn’t how you executed these operations, including a list of the commands used may be useful for those trying to evaluate minor implementation details, either to reproduce a paper’s findings or to apply a similar process to their own data. (It’s also worth noting that running commands using scripts is highly preferable to attempting to reconstruct these commands after the fact—see “Setting up a reproducible project,” above, for other techniques to keep in mind.)
4. **Pipeline specifications and configuration files.** The files defining a series of data-processing steps (sometimes called “pipeline code”) are valuable resources, regardless of how much sophisticated automation they use. Workflow automation tools such as Snakemake [48] or Nextflow [49] can streamline your own bioinformatics work and make it easier to reproduce, but the Bash and Perl scripts used by many still provide valuable documentation of the process, even in situations where the files are written to work only for your data or to run only on a specific machine. Automating the installation and configuration of your tools with workflow managers, package managers (for example, Conda, used for the coordination of installing dependencies), and software container platforms (for example, Apptainer/Singularity [50], Docker) can make things easier for you to manage, but they also make it easier for interested parties to learn about your environment even if they can’t execute the exact code. If you did not use a workflow automation tool, it would still be useful to include a brief summary of all the steps performed to generate your results and figures.
5. **A list of dependencies.** Providing a very specific list of all software dependencies (Glossary) in your pipeline may make a critical difference in how reliably your work can be reproduced. For example, when version 1.16.0 of the popular Python package NumPy—which can be imported into Python scripts to perform many linear algebra operations—was released, the functionality of its matrix multiplication function was unintentionally dramatically changed. This wasn’t fixed until the release of version 1.16.6 nearly a year later [51,52]. There have been dozens of releases since, but a script that worked one way in 2019 may work very differently now—unless you record the version numbers. Placing a call to “sessionInfo()” within R scripts should print all package versions in the output of the script. In Python, running “pip freeze” (or its equivalent, if you’re using a different tool such as Conda for installing packages) will print out the versions of the packages installed in your environment. A software container (Glossary), such as those on the Docker and Singularity platforms, with everything already installed would provide a more complete account of dependencies, but even a list of library versions will cover most contingencies that don’t involve low-level factors such as drivers and differences in hardware [53].
6. **A list of almost-dependencies.** Though a software “dependency” is generally a reference to a package or library that must be locally available for the script to run, there are likely other things your code also depends on. Components of your pipeline may not be “dependencies” per se, but they can be critical to reproducing your work. Reference databases, for example, don’t need to be shared with your code (assuming they are publicly available), but noting the version in your methods section is important because different reference databases can result in disparate results [54], even between minor versions of the same database [55]. Similarly, it is also helpful to note the operating system on which the code was run, particularly if you’re using command-line utilities. Tools can behave differently when moved between platforms, and commands that work on a Linux machine may fail on Windows (or on slightly different distributions of Linux). Even worse, they may finish “successfully” and return different results. One example of this is a popular text-manipulation tool called sed, useful for performing repetitive text substitutions in large files. Multiple versions of sed have been developed for various operating systems, with different approaches for users to define which strings should be altered and in what ways [56]. A script may work as intended on a Linux computer with a specific version of sed [57], but running the same script on a macOS computer may not find the same strings for replacement, or even recognize the same command-line options [58].
7. **New software applications.** If you wrote a whole new program to perform your work, it’s essential to be as transparent as possible about how it works. The editorial staff at the journal should provide guidance about how to handle software that you don’t intend to make open source. Best practices for tool development have been well covered elsewhere [59–64] and are outside the scope of this review, but if you’ve developed a useful tool that is not the primary focus of your work, it may be helpful to submit a separate software paper or “application note” about your program to a computational journal [65], such as the Journal of Open Source Software (https://joss.theoj.org).
8. **A copyright notice and license.** If you are publishing a new computational tool or software package, choosing a license that describes what rights you’ve reserved may play a critical role in its wider adoption because it enables potential users to know which ways of using and sharing your code are legally acceptable. If you have specific needs around allowing (or controlling) reuse, then it would be beneficial to speak with an expert from your institution’s library or office of legal counsel. Even if you are simply sharing the scripts you wrote to clean your data or process images, you can avoid potential headaches, such as emails from individual users asking for permission to modify your code for their work, if you include a “LICENSE.txt” file specifying the copyright holder (possibly your university) and the terms under which others can use, modify and share the code. This is one area in which even prominent companies with legal teams opt for commonly used [66], well-documented licenses such as MIT and Apache 2.0. Journals may have specific recommendations as well, and guides are available from organizations such as the Open Source Initiative (https://opensource.org/licenses) to help you decide.

### Preparing the code

#### Source code is necessary, derivative files aren’t

The most important files to include are the source code files themselves—the files with extensions such as “.py” and “.R” that were written by those performing the analyses. Byproducts of these scripts or their dependencies are not necessary to share—files such as those ending in “.pyc” and those created by the installation of packages, for example, will be regenerated by the user doing their own installations. A simple exercise for distinguishing these files is to try rerunning your code on a machine that was uninvolved in the original analysis: Some files will be regenerated on the new machine, others will need to be downloaded instead, and the ones that you need to move manually are likely the ones most important to share.

#### Test your reproducibility, or ask a friend

Switching workstations can also help highlight aspects of your code that will get in the way of others trying to run it themselves. Removing things like passwords and Application Programming Interface (API; Glossary) keys is critical, but other workstation-specific settings can also cause confusion: Hard-coded file paths will likely only work on a machine connected to that exact file system, even when pointing at common utilities. Storing strings like these in variables declared at the top of your script will provide clues to future users that this is a value they need to specify themselves, or an important variable that is used in multiple places. Showing your work to a colleague may also highlight other sources of confusion in the materials you’re sharing: A person less familiar with the data may not know how to distinguish case samples from controls, for example, or your column labels may not be as intuitive as they seem. There are numerous resources available discussing practical considerations in data curation and sharing [67–72].

#### Rerun inherited code when practical

Another potential reproducibility headache is hiding in code written by people who are no longer contributing to the project—they may not have documented quick fixes or special cases, or they may have used older versions of libraries that have since changed their functionality. If possible, it can be helpful to make sure these scripts still run as expected before sharing. This can also work as an informal form of code review: While you’re working through this code, you may get a better understanding of the data or, in some cases, catch long-forgotten errors.

#### Build bridges over black boxes

You may also encounter situations where there are computational steps that do not have any executable scripts associated with them: Perhaps a critical tool requires a commercial license, or it lacks a programmatic interface, making the “point-and-click” approach using menus and buttons as the only option. In situations like this, document whatever you can. Proprietary sections create a “black box” for future users of your code, in that they can see your input and output of the proprietary section but may lack the resources to run it themselves. It can be helpful to write out the computational steps both before and after the proprietary step and provide a bridge between sections of usable code by indicating where the difficult step is located. Providing users with the output of these tricky steps will enable them to skip over that step and pick up where you left off.

#### Provide documentation

A brief “README” text file should be the minimum descriptor of your code. Providing even a cursory description of how to follow the pipeline could make a big difference in how useful your files are to others, doubly so if you provide more detail about things like important parameters, installing complex dependencies, and where to place input files [26]. Consider someone who has read your paper and is trying to learn more from looking at your code—where should they start? If they want to reproduce an analysis, which script should they run, and which files will the script be looking for? Does anything need to be installed first? This is the file where you can explain in plain language how someone can use your code to answer their questions.

#### Deposit your files somewhere public and permanent

Repository services such as GitHub, GitLab, and Sourceforge are reliable tools for collaboration and distribution [73,74], particularly for ongoing software projects. However, many organizations such as university libraries [75] and groups like the Software Sustainability Institute [76] are skeptical of relying exclusively on commercial enterprises that haven’t explicitly stated no-cost perpetual hosting of digital artifacts as one of their goals. In contrast, repositories such as Zenodo are free, publicly funded projects with decades-long retention plans [77]. Depositing files in a repository that mints DOIs makes it simpler for users to cite the work (and its version) as specifically as possible and makes links less likely to break over time by providing a canonical URL through the DOI resolution service available at doi.org. Deposits with these services are intended to be immutable artifacts, however, so it is preferable to wait until you have a “final” version before sharing there. If you are reluctant to share a permanent version of your data and code prior to publication, many journals (and repositories, such as Zenodo [78] and the Sequence Read Archive [79]) have mechanisms for sharing these resources with reviewers privately first. In addition, Zenodo allows new “versions” of archives to be uploaded, which provides a streamlined way to make corrections or additions if necessary, both before and after publication. There is also a process for linking a GitHub repository to Zenodo [80], if you’re already using GitHub and would like “snapshots” saved in a more citable format.

## Conclusions

Here, we have reviewed almost 3 dozen articles about reproducible research practices to summarize their recommendations. We hope this summary will be useful for those seeking to share their code to comply with requirements from journals or funders, or simply because they are enthusiastic about participating in the open science ecosystem [24]. The factors involved in the sharing of data and code—and in open science issues more broadly—are complex, both for researchers and research subjects, particularly in relation to the equitable participation of marginalized communities [81–84]. If you’ve decided to share your code, these recommendations will provide a starting point for the effort and connections to more detailed sources. Finally, we note that code sharing best practices should be taught early in the science curriculum alongside other Open Science approaches [85–89].
